# Effect of Pyroligneous Acid on Needle Retention and Certain Stress-Related Phytochemicals in Balsam Fir (*Abies balsamea*)

**DOI:** 10.3390/plants15020261

**Published:** 2026-01-15

**Authors:** Niruppama Senthilkumar, Ravalika Kasu, Raphael Ofoe, Lord Abbey, Mason T. MacDonald

**Affiliations:** Faculty of Agriculture, Dalhousie University, Bible Hill, NS B2N 5E3, Canada; nr977159@dal.ca (N.S.); rv260387@dal.ca (R.K.); raphael.ofoe@dal.ca (R.O.); labbey@dal.ca (L.A.)

**Keywords:** abscission, carotenoids, chlorophyll, conifer, flavonoids, membrane injury, phenolics, proline, reactive oxygen species

## Abstract

Balsam fir is an important specialty horticultural crop in eastern North America and commonly harvested for use as Christmas trees. Postharvest quality is a major challenge for producers, who are particularly concerned about postharvest needle retention. It was hypothesized that pyroligneous acid (PA) would help increase postharvest needle retention in balsam fir when supplied via xylem or foliage. This project first identified foliar spraying as the best application method, then designed a multivariate experiment with two factors. The first factor was foliar treatment (control, water, 1% PA, 2% PA, and 4% PA). The second factor was time, where branches were evaluated for needle abscission at 0, 2, 4, 6, and 8 weeks after harvest. The experiment was replicated 5 times and needle abscission, water uptake, chlorophyll, carotenoids, flavonoids, total phenolics, membrane injury, proline, and H_2_O_2_ production were all measured in response. Postharvest abscission reached 100% over the 8-week experiment and water uptake decreased by over 80%. Chlorophyll, proline, membrane injury, and H_2_O_2_ production all increased over time. Although PA did not improve needle retention compared to the control under the tested conditions, 4% PA spray increased proline concentration by 40% while decreasing membrane injury by 26%. Ultimately, PA did not consistently improve needle retention but did induce proline accumulation and membrane protection.

## 1. Introduction

Christmas trees are a culturally and economically important specialty crop. Conifers were used in Christmas celebrations beginning in the 16th century and have since evolved to require millions of trees each year [1]. The Christmas tree industry in Canada alone was worth $163.3 million in 2021 [2]. Larger Christmas tree-producing areas, such as the United States and Europe, have over $1 billion in annual sales [3]. However, postharvest needle retention is a major challenge for the industry, with estimated economic losses exceeding $600 million [4].

Postharvest needle retention is a complex physiological process that presents a major challenge for Christmas tree producers. A multitude of factors have been shown to influence needle retention [5,6,7,8,9,10]. However, the driving factor for postharvest needle abscission appears to be dehydration and/or water uptake deficit through embolism despite the provision of water [11,12,13]. Freshly cut branches uptake water at 0.20 mL g^−1^ d^−1^. However, water uptake, stomatal conductance, and water potential all decrease within the first 2 weeks postharvest, which directly coincides with the commencement of leaf abscission [8,13].

The original premise behind postharvest dehydration was that if water could be conserved by blocking stomata, then dehydration would decrease, and needle retention would increase. However, anti-transpirants have generally been only effective at reducing transpiration, with little effect on water status and needle retention [14,15]. Another strategy was delivering water to trees via an intravenous type of device, which was also ineffective [16]. Several species face postharvest challenges in water uptake due to bacterial accumulation in the water and xylem of the cut end [13,17]. Xylem blockages (embolism) can be mitigated to some extent by antibacterial leachates from the plant into the water, practices such as changing water daily, or addition of low-concentration silver nitrate to standing water [13,18]. However, such strategies are inconvenient and not guaranteed to work.

Pyroligneous acid (PA) is a by-product of pyrolysis where plant biomass undergoes thermal degradation in the absence or near absence of oxygen. The condensed organic vapors form an aqueous liquid fraction, PA, that has several desirable properties [19]. PA has been an effective organic biostimulant, fertilizer, insecticide, fungicide, bactericide, and antioxidant [20,21,22,23]. As such, PA has effectively increased growth, maintained chlorophyll membranes, reduced the impact of stress, and inhibited fungal and bacterial growth in agricultural species. Furthermore, the use of PA in agriculture is desirable because it can be produced sustainably and has virtually no environmental footprint [24,25]. The combination of PA’s beneficial properties has high potential to improve postharvest characteristics of balsam fir, specifically by inhibiting growth of contaminants as a bactericide/fungicide and membrane protection as an antioxidant. It is hypothesized that PA could inhibit bacterial blockage in balsam fir xylem, inhibit fungal contamination of stomata, and protect chloroplast membranes. The specific objectives of this research are to determine (1) the effectiveness of xylem-fed PA on balsam fir needle retention, (2) the effectiveness of foliar applied PA on balsam fir needle retention, and (3) any effect on phytochemical composition. Although this study investigates physiological responses only and does not test antifungal or antibacterial mechanisms directly, all the three hypothesized mechanisms could lead to improved postharvest needle retention.

## 2. Results

### 2.1. Experiment 1: Determination of Application Method and Concentration Range

#### 2.1.1. Xylem-Fed Delivery of PA

There was a significant (*p* < 0.001) difference in all water quality response variables due to PA concentration (Table 1). The significant differences in water quality parameters, specifically pH, were expected since PA is an acidic aqueous solution. There was considerable variation in pH, salinity, total dissolved solids (TDS), and electrical conductivity (EC). It is noteworthy that there is a clear trend that pH greatly decreases with even a small percentage of PA, while salinity, TDS, and EC all increase.

**Figure 1:**
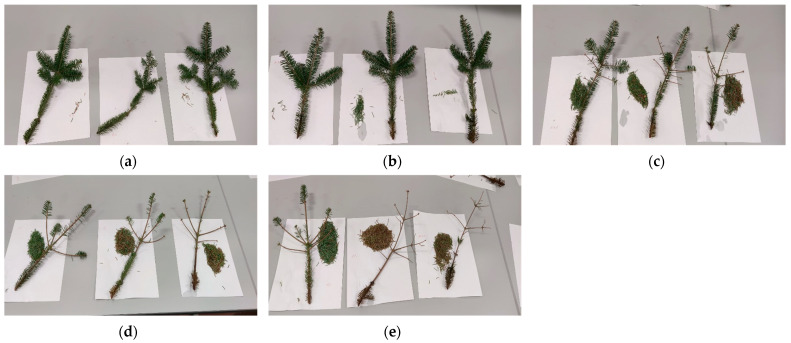
Visual inspection of branches after finger run test when displayed in (**a**) 0%, (**b**) 0.25%, (**c**) 0.5%, (**d**) 1%, or (**e**) 2% pyroligneous acid after 6 weeks of xylem feeding.

Visual differences in abscission due to xylem uptake are shown in Figure 1. There was a significant (*p* < 0.001) difference in needle abscission due to PA concentration (Table 1), but not the trend we had expected. The 0.25% PA solution had no impact on abscission compared to the control, but every other concentration significantly (*p* < 0.05) increased abscission. It is possible that PA altered water chemistry too much at concentration above 0.25%. The pH was very low compared to distilled water, while salinity, TDS, and EC had all significantly increased.

#### 2.1.2. Foliar Application of PA

**Figure 2:**
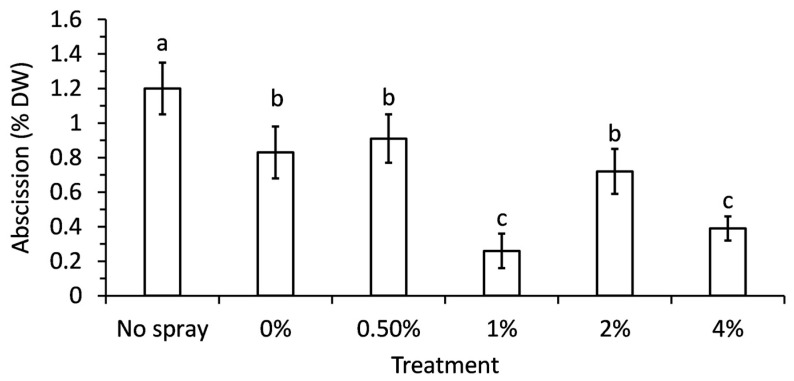
Needle abscission percentage in balsam fir seedlings after foliar spray with various concentrations of pyroligneous acid after 8 weeks. Bars represent the means, and the error bars indicate standard error. Each mean was calculated from 5 replicates from independent experiments and those bars with different letter groupings are significantly different (*p* < 0.05).

There was a significant difference (*p* = 0.035) in needle abscission due to foliar spray treatments (Figure 2). The no-spray control lost significantly more needles than any other treatment, while 1% and 4% PA sprays lost significantly less needles. The 1% PA treatment lost 78% fewer needles than the no-spray control and 69% fewer needles than the water-only spray. The 4% PA treatment lost 66% fewer needles than the no-spray control and 55% fewer needles than the water-only spray. However, it is also important to state that the raw numbers are all very small percentages of the total needles. Ultimately, all branches retained needles reasonably well throughout the 5 weeks of this experiment, but the 1% and 4% PA foliar treatments were statistically the best. The 1%, 2%, and 4% treatments were selected for future experiments.

### 2.2. Experiment 2: Effect of Foliar PA on Postharvest Needle Retention

**Figure 3:**
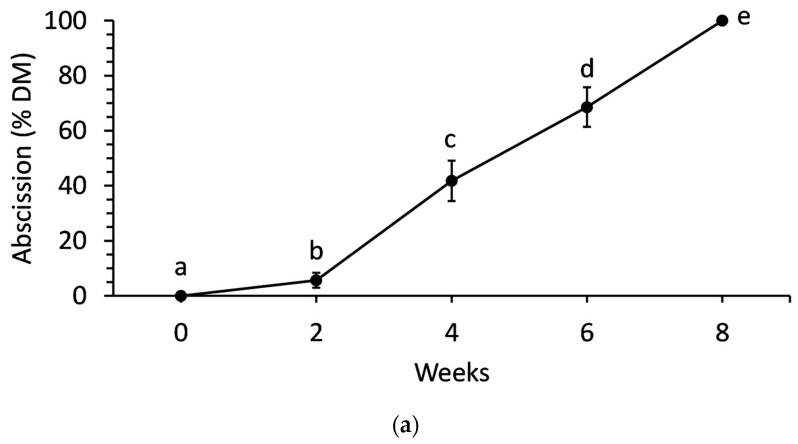

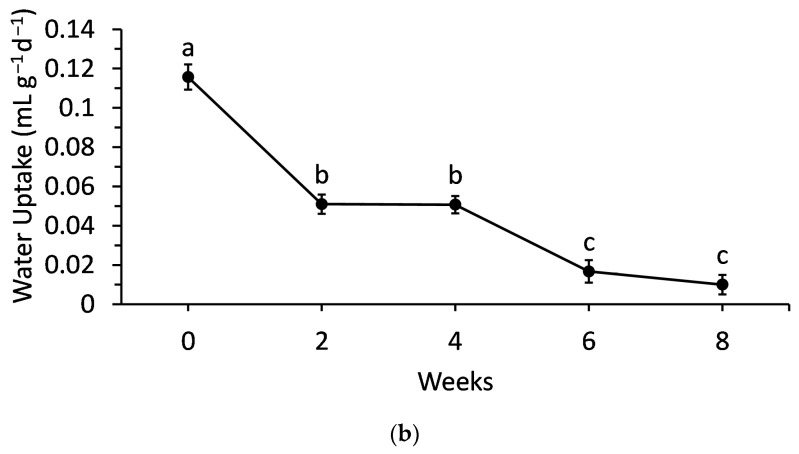
(**a**) Needle abscission and (**b**) water uptake of balsam fir branches over time. In each, data points denote the means and error bars denote standard error from a pooled 25 branches. Since treatment effects were not significant, displayed means are pooled from all treatments and show only time effects. Means with different letters are significantly different as determined by Fisher’s least significant difference at 5% significance.

There were no significant differences in needle abscission or water uptake due to foliar PA treatments (*p* = 0.961 and *p* = 0.230, respectively) when branches were monitored until complete abscission. However, needle abscission and water uptake each increased significantly (*p* < 0.001) over time (Figure 3). When data from PA treatments was pooled to clearly depict time effects, needle abscission began slowly with only 5% of needles abscised within the first 2 weeks. Needle abscission then accelerated, increasing 37% over the next 2 weeks. Abscission continued to 100% over the remaining 4 weeks almost linearly. Meanwhile, water uptake decreased rapidly in the first 2 weeks by 56% (Figure 3). Water uptake did not decrease significantly again until week 6.

### 2.3. Experiment 3: Effect of Foliar PA on Stress Response

**Figure 4:**
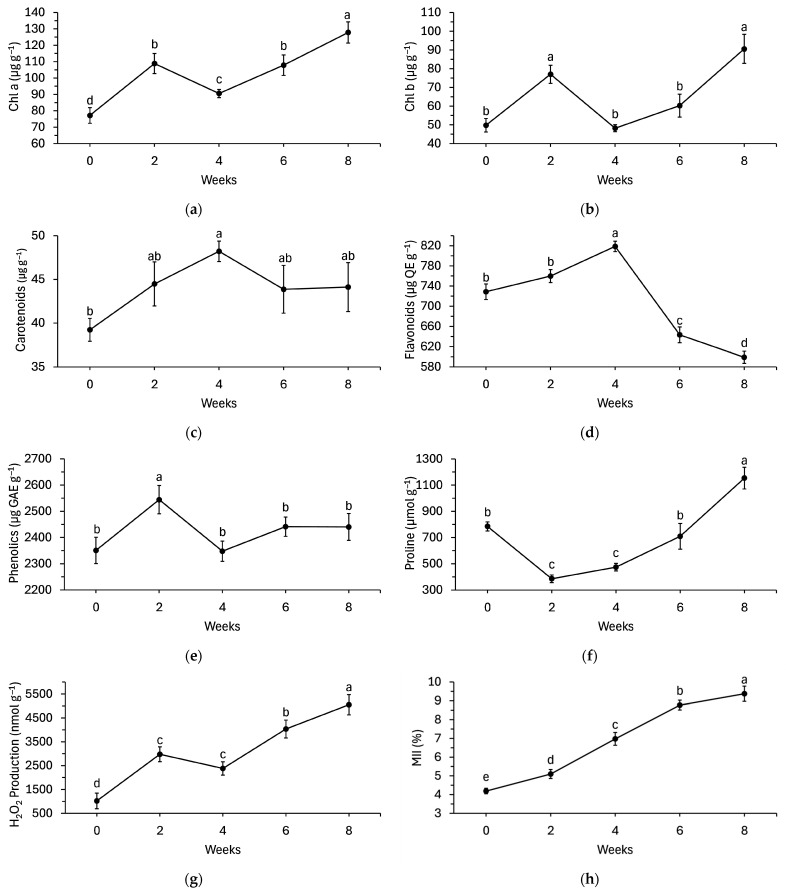
(**a**) Chlorophyll a, (**b**) chlorophyll b, (**c**) carotenoids, (**d**) flavonoids, (**e**) phenolics, (**f**) proline, (**g**) H_2_O_2_ production, and (**h**) membrane injury of balsam fir branches over time. In each, data points denote the means and error bars denote standard error from a pooled 25 branches. Time effects were pooled across control, water, 1% PA, 2% PA, and 4% PA treatments. Means with different letters are significantly different as determined by Fisher’s least significant difference at 5% significance.

All pigments significantly (*p* < 0.001) changed over time throughout the experiment (Figure 4). Chl a and chl b increased by 41% and 55%, respectively, within the first 2 weeks before decreasing to near initial concentrations by week 4 (Figure 4a,b). Chl a and Chl b increased throughout the rest of the experiment and ultimately 66% and 82% higher than initial concentrations, respectively. Carotenoid concentration had a different trend, where it increased by 23% by week 4 from initial concentrations. The carotenoid concentration decreased for the rest of the experiment to a level not significantly different than the initial concentration (Figure 4c).

Flavonoids and total phenolics significantly changed over time (*p* < 0.001 and *p* = 0.031, respectively). Flavonoids increased by 12% by week 4 compared to initial concentrations, but then rapidly decreased (Figure 4d). By week 8, flavonoids were 18% lower than initial concentrations. Total phenolics only increased during the second week but otherwise did not differ significantly from initial concentrations (Figure 4e).

Stress indicators included proline, H_2_O_2_ production, and membrane injury index and all eventually increased by the end of the experiment. Proline was originally present at 785.0 µmol g^−1^ but decreased by 51% in the first 2 weeks (Figure 4f). Proline concentration recovered by week 6 before increasing by 47% by week 8. H_2_O_2_ production and membrane injury followed a more linear progression over time. H_2_O_2_ production increased 191% by week 2, 133% by week 4, 295% by week 6, and 395% by week 8 compared to initial levels (Figure 4g). The membrane injury index increased 22% by week 2, 66% by week 4, 109% by week 6, and 124% by week 8 compared to initial levels (Figure 4h).

There was a significant foliar spray treatment effect on chl a and chl b (*p* = 0.001 for each), where the water-sprayed treatment had significantly lower chlorophyll (Table 2). Chl a and chl b were 18% and 33% lower than the control, respectively. Chlorophyll did not differ from the control in any PA spray treatment. Similarly, total phenolics were 14% lower in the water-sprayed branches compared to the control, but spraying with PA had no significant effect (Table 2). Carotenoids and flavonoids were not significantly different in any treatment (*p* > 0.05).

There was a significant treatment effect on proline (*p* = 0.014). Water-sprayed branches had 19% less proline than the control, while branches sprayed with 4% PA had 40% more proline than the control (Table 3). There was a significant treatment effect on H_2_O_2_ production (*p* = 0.032), though the effect was only observed in one treatment. Water-sprayed branches had 36% less H_2_O_2_ production (Table 3). There was also a significant treatment effect on the membrane injury index (*p* < 0.001). Membrane injury was 6% lower in the 1% PA treatment, 29% lower in the 2% PA treatment, and 26% lower in the 4% PA treatment compared to the control (Table 3).

## 3. Discussion

### 3.1. PA and Needle Retention

The progression of needle abscission and decline in water uptake observed in this study mirrors established patterns for balsam fir and other conifer species. As shown in Figure 3a, needle abscission was minimal in the first two weeks postharvest, accelerated sharply between weeks two and six, and reached completion by the eighth week. Water uptake (Figure 3b) dropped significantly within the first two weeks, then stabilized at low levels as abscission increased. These findings are consistent with previous studies [8], that highlights dehydration and xylem embolism as key drivers of postharvest needle loss. Like earlier studies, this current study confirms that water status is a critical determinant of needle retention in balsam fir, with water uptake deficits directly preceding and likely triggering abscission. These findings suggest that xylem dysfunction and embolism formation can occur on the branch and whole-tree level under stressed conditions and that future work should consider the effects of these conditions on whole trees under commercial postharvest conditions.

### 3.2. PA Effects on Pigments and Antioxidant Metabolites

All major pigments and stress-related phytochemicals changed significantly over time in balsam fir branches (Figure 4). Chlorophyll a and b increased in the first two weeks, then declined near baseline by week four and ultimately rose again above initial levels by week eight. Carotenoids peaked in week four before returning to baseline. These results align with other species, where PA showed a positive effect in increasing chlorophyll pigment in tomato leaves [26] and Indian mustard [26]. This increase in chlorophyll pigments may suggest that PA could have delayed chlorophyll degradation by suppressing senescence-related pathways [27] or by providing antioxidant compounds that stabilize pigment metabolism [28]. Interestingly, the PA used in this study was shown to contain high levels of N, K and Ca as well as other bioactive compounds including phenolics and flavonoids [29]. Also, in this study, flavonoids and phenolics showed transient increases, with flavonoids peaking at week four and dropping below initial levels by week eight. While these increased phenolic compounds are fundamental for maintaining metabolic stability by scavenging ROS, their levels have also been associated with improved chlorophyll contents in leaf tissues by suppressing oxidative stress induced photorespiration [30]. Hence, the increased chlorophyll content with PA application can be ascribed to its rich bioactive contents that modulated pigment breakdown, possibly via improved chlorophyll biosynthesis and reduction in oxidative stress damage.

Proline, H_2_O_2_ production, and membrane injury all increased over the course of the experiment. These trends differ from those in other species. In apples and tomatoes, PA treatments led to sustained increases in antioxidant capacity and suppression of ROS, delaying abscission and reducing the postharvest losses [31,32]. In this experiment, however, ROS and membrane injury increased steadily despite the increase in antioxidants like proline and flavonoids, suggesting that PA did not mitigate oxidative stress. This may reflect species-specific differences in cuticle structure, stress signaling or the relative importance of hydraulic versus biochemical factors in abscission.

Branches treated with 4% PA had more proline than controls, while water-sprayed branches had less (Table 3). Proline is a well-established osmoprotectant, accumulating in plant tissues under drought and other abiotic stresses [33]. Increased proline here indicates that PA may have triggered a stress response, potentially aiding in osmotic adjustment and temporary stabilization of cellular structures. However, proline accumulation alone was not sufficient to prevent needle loss, likely because the primary cause of abscission was hydraulic failure rather than cellular dehydration [34]. This apparent coexistence of both increased proline and hydraulic failure represents a layered stress physiology where proline accumulation reflects a short-term cellular response that may delay visible senescence but cannot prevent the structural failure of the vascular system. PA treatments reduced membrane injury compared to controls (Table 3) suggesting that PA may offer some protection to cellular membranes, possibly by enhancing antioxidant defenses or stabilizing lipid bilayers [35,36]. In other crops, such as apples, similar effects are associated with delayed senescence and improved postharvest quality [31]. A study in canola seed showed that PA has a positive effect in increasing the SOD activity and reducing oxidative stress [36]. However, in balsam fir, these improvements did not translate into better needle retention, again highlighting the dominant role of hydraulic factors over biochemical stress responses in this species.

### 3.3. Hydraulic Versus Biochemical Control of Needle Abscission

Even though PA increased proline and reduced membrane injury, needle retention was not consistently improved by PA because of several reasons. First, needle abscission in balsam fir is driven primarily by water uptake deficits and xylem embolism [34]; improvements in cellular stress tolerance could not overcome systemic hydraulic failure. Second, while not directly measured in this study, ethylene is a known trigger of abscission in conifers [4,34,37,38]. PA may not have affected ethylene signaling pathways or may have encouraged ethylene production. While PA can modulate biochemical stress markers, these are not the primary determinants of abscission in balsam fir, unlike in some angiosperms, where oxidative stress is more central to senescence and tissue loss [39]. Finally, conifer needles have a thick, waxy cuticle [40], which likely limited PA penetration and efficacy. Unlike apples and tomatoes, PA may not have reached the target tissues in balsam fir.

This study shows that whereas PA can modulate biochemicals linked to stress and decrease membrane damage in balsam fir, these modifications do not result in better needle retention in postharvest settings. The results highlight the significance of hydraulic parameters in conifer abscission and recommend that future research concentrate on methods that directly address embolism and water intake. Also, since light conditions were not controlled, the influence of photoperiod and light quality on postharvest physiology could not be assessed. Future studies should examine these factors under defined photoperiods. Future research may provide more encouraging outcomes if alternative surfactants are used to improve PA penetration, replication, and combinatorial treatments that target both physiological and biochemical processes.

## 4. Materials and Methods

### 4.1. Experiment 1: Determination of Application Method and Concentration Range

#### 4.1.1. Sample Collection

Branches were collected from the Christmas tree clonal orchard in Plumdale Research Facility at Dalhousie University. Branches were cut to include 2 full years of growth at a height of 1 m. Branches were first visually inspected and any with obvious signs of stress (e.g., nutrient stress discoloration, pest damage, etc.) were excluded from the study. A total of 55 branches were required for the first experiment, so 5 branches were collected from 11 trees of the same genotype within the clonal orchard.

Samples were taken to the lab, provided a fresh cut approximately 1 cm from the previously cut end, and then placed in glass vases to simulate household conditions. All cuts were performed with the cut section submerged in deionized water to reduce the risk of introducing air bubbles to trigger embolism. Branches were then randomly assigned to treatments via a random number generator.

#### 4.1.2. Xylem-Fed Delivery of PA

This experiment followed a completely randomized design where the factor of interest was PA concentration diluted in deionized water. Treatment levels consisted of 0% (a water-only control), 0.25%, 0.5%, 1%, and 2% PA solutions. Each branch was provided with 1 L of its respective treatment solutions. The base of each branch was wrapped in aluminum foil at the mouth of the vase to reduce evaporation and provide stability to the branch. Since PA is a naturally derived product containing multiple organic compounds, specific water quality parameters pH, salinity, total dissolved solids (TDS), and electrical conductivity (EC) of solutions were measured at the beginning of the experiment. This experiment was replicated 5 times.

#### 4.1.3. Foliar Application of PA

This experiment followed a completely randomized design where the factor of interest was PA concentration. Treatment levels consisted of a control (no spraying), 0% (water only spray), 0.5% PA, 1% PA, 2% PA, and 4% PA. Each branch was provided with 1 L of deionized water, and the base of the branch was wrapped in aluminum foil at the mouth of the vase to reduce evaporation and provide stability to the branch. Branches were sprayed with their respective treatment at the beginning of the experiment and then once per week throughout the remainder of the experiment. The experiment was conducted under ambient laboratory conditions at room temperature in typical indoor lighting with 40% relative humidity. This condition was selected to represent a worst-case scenario of water stress, thereby allowing us to evaluate treatment efficacy under challenging circumstances. It is worth noting that no controlled photoperiod, artificial light/dark cycle, or supplemental light sources were applied. As above, spray solutions were evaluated for pH, salinity, TDS, and EC. This experiment was replicated 5 times.

#### 4.1.4. Measuring Needle Abscission

Since these experiments were preliminary in nature, the major response variable was only needle abscission. Needle abscission was determined by performing a finger run test [5,11]. The first finger run test for both experiments was conducted on 4 January 2023. The water additive experiment was terminated at this point. A second finger run test was conducted 23 January 2023, on the remaining foliar spray experiment, after which the foliar spray experiment was also terminated. In each case, abscised needles and branches were collected, dried in an oven at 80 °C for 24 h, and then weighed. Needle abscission was then expressed a percentage of total dry mass of a branch.

Data from each experiment was submitted to an analysis of variance to determine significant differences. The null hypothesis was that there was no difference between treatments, and the alternative was that there was at least one difference. Specific differences were then determined through Fisher’s least significant difference at 5% significance. Statistical assumption of normality, homogeneity, and independence were all valid.

### 4.2. Experiment 2: Effect of Foliar PA on Postharvest Needle Retention

#### 4.2.1. Experimental Design

This experiment had two factors. The first factor was foliar treatment, which included a control (no spraying), water, 1% PA, 2% PA, and 4% PA. The second factor was time, where branches were evaluated for needle abscission at 0, 2, 4, 6, and 8 weeks after harvest. The experiment was replicated 5 times. Branch collection and display protocol were the same as Experiment 1. Needle abscission and water uptake were the only response variables. Experiment 2 required 25 branches, where 5 branches were collected from 5 trees and then randomly assigned to treatments.

#### 4.2.2. Needle Abscission

Needle abscission was measured gravimetrically after a finger run test, like Experiment 1. Needles were dried in an oven at 80 °C for 24 h. Branches were displayed until complete abscission occurred, and all branches had completed abscission within the 8-week experiment duration. All needles were weighed at the end of the experiment to determine the total dry mass of needles on each branch. Needle abscission was then expressed as a cumulative percentage.

#### 4.2.3. Water Uptake

Water uptake was determined gravimetrically as suggested by Lada et al. [13]. To determine water uptake the branches were gently removed from the amber flasks and the remaining contents (water and flask together) were weighed every alternate day. Since bottles were sealed, any loss in mass indicated water uptake by the branches. The water uptake was reported in mL g^−1^ d^−1^.

#### 4.2.4. Statistical Analysis

The data collected from the experiment was analyzed statistically using Minitab 22 software (Minitab, LLC, State College, PA, USA). Analysis of variance (ANOVA) from the general linear model was used to determine if there is a difference in needle retention or water uptake due to foliar PA in balsam fir. The residuals from needle retention data were nearly normal (*p* = 0.034) from an Anderson-Darling test; normality was achieved with a mild square root transformation. Mean separation was determined using Fisher’s method with a 95% confidence interval at *p* ≤ 0.05 after a significant ANOVA result was obtained.

### 4.3. Experiment 3: Effect of Foliar PA on Stress Response

#### 4.3.1. Experimental Design

Like Experiment 2, this experiment had two factors. The first factor was foliar treatment, which included a control (no spraying), water, 1% PA, 2% PA, and 4% PA. The second factor was time, where branches were evaluated for needle abscission at 0, 2, 4, 6, and 8 weeks after harvest. Display conditions were identical to those described earlier in Experiment 1. The branches used in Experiment 3 were distinct from those used in previous experiments. and the experiment was replicated 6 times. The destructive nature of sampling for biochemical analysis required all needles on each branch to be removed every 2 weeks, ensuring that biweekly sampling was independent As such, 150 branches were needed for Experiment 3, which were obtained by collecting 15 branches from 10 trees.

Response variables were chlorophyll a, chlorophyll b, total carotenoids, total phenols, total flavonoids, proline concentration, reactive oxygen species, and membrane injury index.

#### 4.3.2. Chlorophyll and Carotenoids

Chlorophyll a, b and carotenoids were measured in reference to Lichtenthaler [41]. A 0.2 g of ground samples were transferred into a sterile 50 mL falcon tube and 10 mL of 80% acetone was added. The mixture was vortexed for 1 min and centrifuged at 12,000× *g* for 15 min. 1 mL of supernatant was transferred into a cuvette. The absorbance was measured at 646.8 and 663.2 nm with a UV-Vis spectrophotometer against 80% acetone as blank. The concentration of chlorophyll a, b, and carotenoids was calculated as µg g^−1^ FW using the Formulas (1)–(3).

(1) $$Chlorophyll\;a\left(\frac{\mu g}{mL}\right)=(12.25\times A663.2)-(2.79\times A646.8)$$

(2) $$Chlorophyll\;b\left(\frac{\mu g}{mL}\right)=(21.50\times A646.8)-(5.1\times A663.2)$$

(3) $$Carotenoids\left(\frac{\mu g}{mL}\right)=\frac{\left(1000\times A470)-(1.8\times chl\;a)-(85.02\times chl\;b\right)}{198}$$

#### 4.3.3. Phenolics and Flavonoids

The total phenolic content was determined by the Folin–Ciocalteu assay described by Ainsworth & Gillespie [42] with a slight modification. A 0.2 g of sample was homogenized in a 2 mL of ice-cold methanol and incubated in the dark at room temperature for 48 h. The mixture was centrifuged at 13,000× *g* for 5 min and 100 μL of supernatant was transferred into a new microfuge tube 200 µL of 10% Folin-Ciocalteau reagent was added and vortexed for 5 min. 800 µL of 700 nM sodium carbonate (Na_2_CO_3_) was added, vortexed for a min, and incubated at room temperature (25 °C) for 2 h. The absorbance of the resultant mixture was measured at 765 nm. Total phenolic content was estimated using gallic acid equivalents standard curve and was expressed as mg gallic acid equivalents per g of the sample.

Total flavonoids was determined as described by Chang et al. [43] with some modifications. A 0.2 g of ground samples were homogenized with 2.5 mL of 95% methanol. The mixture was centrifuged at 13,000× *g* for 10 min and 500 µL of the supernatant was transferred into a new tube. To each tube, 1.5 mL of 95% methanol, 0.1 mL of 10% AlCl_3_, 0.1 mL of 1 M potassium acetate, and 2.8 mL of distilled water was added. The mixture was vortexed and incubated for 30 min at room temperature and absorbance was measured at 415 nm against a blank. Flavonoids content was estimated using a quercetin standard curve and expressed in µg of quercetin per g of sample.

#### 4.3.4. Proline

A 0.5 g of ground samples were mixed with 1 mL of 70% ethanol. Then the mixture was centrifuged at 12,000× *g* for 15 min at 4 °C and 500 µL of supernatant was added to 1 mL of the reaction mixture (ninhydrin 1% (*w*/*v*) dissolved in 60% acetic acid (*v*/*v*) and 20% ethanol (*v*/*v*). The tubes were sealed and vortexed for 30 s. The mixture was incubated in a water bath at 95 °C for 30 min. After cooling at room temperature, the absorbance was measured at 520 nm using a spectrophotometer against a blank containing ethanol and reaction mixture. Proline content was estimated using the L-Proline standard curve. The absorbance was plotted against L-Proline concentration to obtain the standard curve. Total proline content can be calculated using Equation (4).

(4) $$Proline\left(\frac{\mu mol}{gFW}\right)=\frac{{Abs}_{ext}-blank}{Slope}\times {Vol}_{ext}/{Vol}_{aliquot}\times \left(\frac{1}{FW}\right)$$

In Equation (4), where Abs_ext_ is the absorbance of plant extract, blank is the absorbance of clear extraction solution, the slope is determined by linear regression of a calibration curve, Vol_ext_ is the total extract volume, Vol_aliqout_ is the extract volume used for the assay, FW is the weight of the plant material.

#### 4.3.5. Reactive Oxygen Species

ROS was estimated to use the H_2_O_2_ concentration by following the procedure described by Patterson et al. [44]. A total of 0.1 g of ground samples were mixed with 1 mL of 0.1% (*w*/*v*) TC. The mixture was vortexed and centrifuged at 16,000 rpm for 10 min. After that 0.2 mL of supernatant was added to 200 μL of 100 mM potassium phosphate buffer (pH 7.0) and 800 μL of 1 M KI. The mixture was incubated in the dark for 1 h. The absorbance was measured at 390 nm against the blank (0.1 TCA in the absence of needle extract). The H_2_O_2_ concentration was calculated according to the standard curve (H_2_O_2_ dissolved in 0.1% TCA) and expressed in μM/g of fresh weight.

#### 4.3.6. Membrane Injury Index

MII was performed in reference to the study by MacDonald and Lada [8]. The percentage of electrolyte that leaks into the solution was used by the membrane injury index (MII) to measure membrane integrity. Approximately 30 mL of distilled water was added to test tubes, which were then let to warm up to room temperature (25 °C). Using a CDM 2e Conductivity Meter (Vernon Hills, IL, USA), the electrical conductivity of the distilled water (EC_w_) alone was measured. Then, 0.4 g of needles were taken from each branch and immersed entirely in a centrifuge tube. After being sealed, the tubes were kept at room temperature for 24 h. To estimate how much of the electrolytes are leached into the solution, the initial conductivity (EC_0_) was measured. After 4 h at 90 °C in a forced-air oven to kill tissues, sealed tubes were then cooled to room temperature. To estimate the maximum leakage, final conductivity measurements (EC_f_) were made after the system had reached equilibrium at 25 °C. The following formula was used to determine MII.

(5) $$MII={EC}_{0}-{EC}_{w}/{EC}_{f}-{EC}_{w}\times 100$$

#### 4.3.7. Statistical Analysis

Data were submitted to general linear model using Minitab 22 (Minitab, LLC, State College, PA, USA). Foliar application and time were entered as explanatory variables and a 2-way interaction between spraying and time was also included in the model. Means were compared using Fisher’s least significant difference when significant differences were found from the ANOVA at *p* ≤ 0.05. Statistical assumptions of normality, homogeneity, and independence were confirmed.

## Figures and Tables

**Table 1:** Water quality parameters and abscission due to PA added to stand water. Data is expressed as mean ± standard error. Different letters within columns indicate significant differences between treatments at 5% significance using Fisher’s least significant difference test.

Concentration (%)	pH	Salinity (ppm)	TDS (ppm)	EC (µS)	Abscission (%)
0	6.15 ± 0.09 A	441.0 ± 0.6 A	634.7 ± 1.2 A	908.3 ± 1.3 A	1.0 ± 0.5 A
0.25	3.54 ± 0.06 B	490.0 ± 1.2 B	703.4 ± 1.2 B	1008.3 ± 1.76 B	1.3 ± 0.5 A
0.50	3.29 ± 0.02 C	505.7 ± 0.3 C	724.7 ± 0.9 C	1035.3 ± 0.3 C	44.7 ± 5.7 B
1.00	3.23 ± 0.03 C	550.7 ± 0.9 D	784.7 ± 0.7 D	1122.0 ± 1.0 D	67.9 ± 9.5 C
2.00	3.06 ± 0.01 D	577.0 ± 0.6 E	821.0 ± 1.0 E	1174.7 ± 2.3 E	75.2 ± 9.9 C
*p*-value	<0.001	<0.001	<0.001	<0.001	<0.001

**Table 2:** Chlorophyll pigment and stress-related phytochemicals of balsam fir branches due to PA added to the stand water. Data is expressed as mean ± standard error. Different letters within columns indicate significant differences between treatments at 5% significance.

Treatment	Chl a	Chl b	Carotenoids	Flavonoids	Phenolics
	(µg g^−1^)	(µg g^−1^)	(µg g^−1^)	(µg GAE g^−1^)	(µg QE g^−1^)
Control	104.7 ± 7.0 A	73.3 ± 8.8 A	41.2 ± 2.7	737.7 ± 22.0	2493.2 ± 71.4 A
Water	85.4 ± 2.7 B	45.9 ± 1.8 B	46.3 ± 1.4	718.3 ± 21.1	2132.8 ± 87.4 B
1% PA	103.5 ± 6.3 A	63.9 ± 5.2 A	46.1 ± 2.4	704.1 ± 18.7	2546.3 ± 116.1 A
2% PA	111.1 ± 6.9 A	75.1 ± 8.6 A	43.0 ± 3.1	692.0 ± 18.1	2550.9 ± 82.7 A
4% PA	107.3 ± 6.5 A	68.1 ± 7.0 A	43.1 ± 1.4	699.7 ± 18.9	2407.2 ± 85.8 A
*p*-value	0.001	0.001	0.55	0.357	<0.001

**Table 3:** Stress marker contents of balsam fir branches due to PA added to the stand water. Data is expressed as mean ± standard error. Different letters within columns indicate significant differences between treatments at 5% significance.

Treatment	Proline	H_2_O_2_ Production	MII
	(µmol g^−1^)	(nmol g^−1^)	(%)
Control	705.5 ± 102.3 B	3465.5 ± 448.8 A	7.8 ± 0.6 A
Water	570.0 ± 49.3 C	2224.8 ± 322.5 B	8.2 ± 0.6 A
1% PA	610.7 ± 61.2 BC	3302.8 ± 504.0 A	7.3 ± 0.5 B
2% PA	611.4 ± 44.2 BC	3617.0 ± 663.9 A	5.5 ± 0.4 C
4% PA	991.2 ± 122.9 A	2903.2 ± 329.6 AB	5.8 ± 0.3 C
*p*-value	0.035	0.038	<0.001

## Data Availability

The datasets presented in this article are not readily available because the data are part of an ongoing study. Requests to access the datasets should be directed to Mason MacDonald (mason.macdonald@dal.ca).

## Abbreviations

The following abbreviations are used in this manuscript:

EC	Electrical conductivity
MII	Membrane injury index
PA	Pyroligneous acid
ROS	Reactive oxygen species
TDS	Total dissolved solids

## References

[1] Albers H.H., Davis A.K. (1997). The Wonderful World of Christmas Trees.

[2] Garside M. Farm Cash Receipts from Christmas Trees Canada. https://www.statista.com/statistics/449337/farm-cash-receipts-of-christmas-trees-canada/.

[3] Chastagner G.A., Benson D.M. (2000). The Christmas Tree: Traditions, Production, and Diseases. Plant Health Prog..

[4] Thiagarajan A., MacDonald M.T., Lada R. (2016). Environmental and Hormonal Physiology of Postharvest Needle Abscission in Christmas Trees. Crit. Rev. Plant Sci..

[5] MacDonald M.T., Lada R.R., MacDonald G.E., Caldwell C.D., Udenigwe C.C. (2023). Changes in Polar Lipid Composition in Balsam Fir during Seasonal Cold Acclimation and Relationship to Needle Abscission. Int. J. Mol. Sci..

[6] Bartas M. (2024). Abiotic Stresses in Plants: From Molecules to Environment. Int. J. Mol. Sci..

[7] Amponsah I.G., Comeau P.G., Brockley R.P., Lieffers V.J. (2005). Effects of Repeated Fertilization on Needle Longevity, Foliar Nutrition, Effective Leaf Area Index, and Growth Characteristics of Lodgepole Pine in Interior British Columbia, Canada. Can. J. For. Res..

[8] MacDonald M.T., Lada R.R. (2014). Biophysical and Hormonal Changes Linked to Postharvest Needle Abscission in Balsam Fir. J. Plant Growth Regul..

[9] Addicott F.T. (1968). Environmental Factors in the Physiology of Abcission. Plant Physiol..

[10] Manter D.K., Bond B.J., Kavanagh K.L., Stone J.K., Filip G.M. (2003). Modelling the Impacts of the Foliar Pathogen, Phaeocryptopus Gaeumannii, on Douglas-Fir Physiology: Net Canopy Carbon Assimilation, Needle Abscission and Growth. Ecol. Model..

[11] Chastagner G.A., Riley K.L. (2003). Postharvest Quality of Noble and Nordmann Fir Christmas Trees. HortSci.

[12] Tremblay J.D., Smith R.F., D’Orangeville L. (2023). Integrating the Rate of Moisture Loss into Needle Retention Testing to Improve the Selection of Balsam Fir (*Abies balsamea*) for Use as Christmas Trees. Forests.

[13] Lada R.R., MacDonald M.T., West R.R. (2014). Physiology of Postharvest Needle Abscission in Balsam Fir: Water Quality Modulates Postharvest Needle Abscission. Acta Hortic..

[14] Hinesley L.E., Snelling L.K., Goodman S. (1993). “Crop-Life” Does Not Slow Postharvest Drying of Fraser Fir and Eastern Red Cedar Christmas Trees. HortSci.

[15] Duck M.W., Cregg B.M., Cardoso F.F., Fernandez F.T., Behe B.K., Heins R.D. (2003). Can Antitranspirants Extend the Shelf-Life of Tabletop Christmas Trees. Acta Hortic..

[16] Chastagner G.A., Hinesley E., Riley K. (2007). Effectiveness of I–V Watering Devices in Maintaining Postharvest Freshness and Quality of Cut Christmas Trees. Postharvest Biol. Technol..

[17] Hoogerwerf A., Van Doorn W.G. (1992). Numbers of Bacteria in Aqueous Solutions Used for Postharvest Handling of Cut Flowers. Postharvest Biol. Technol..

[18] Hinesley L.E., Snelling L.K. (1995). Postharvest Drying of Leyland Cypress, Eastern Red Cedar, and Fraser Fir Christmas Trees. HortSci.

[19] Mathew S., Zakaria Z.A. (2015). Pyroligneous Acid—The Smoky Acidic Liquid from Plant Biomass. Appl. Microbiol. Biotechnol..

[20] Grewal A., Abbey L., Gunupuru L.R. (2018). Production, Prospects and Potential Application of Pyroligneous Acid in Agriculture. J. Anal. Appl. Pyrolysis.

[21] Lee S.H., H’ng P.S., Lee A.N., Sajap A.S., Tey B.T., Salmiah U. (2010). Production of Pyroligneous Acid from Lignocellulosic Biomass and Their Effectiveness Against Biological Attacks. J. Appl. Sci..

[22] Loo A., Jain K., Darah I. (2008). Antioxidant Activity of Compounds Isolated from the Pyroligneous Acid, Rhizophora Apiculata. Food Chem..

[23] Abinandan S., Kuppan P., Venkateswarlu K., Mukunthan K., Megharaj M. (2025). Pyroligneous Acid as a Multifunctional Biostimulant Enhances Microalgal Growth and Soil Beneficial Metabolites for Sustainable Agriculture. World J. Microbiol. Biotechnol..

[24] Jindo K., Goron T.L., Kurebito S., Matsumoto K., Masunaga T., Mori K., Miyakawa K., Nagao S., Tokunari T. (2022). Sustainable Plant Growth Promotion and Chemical Composition of Pyroligneous Acid When Applied with Biochar as a Soil Amendment. Molecules.

[25] Mmojieje J., Hornung A. (2015). The Potential Application of Pyroligneous Acid in the UK Agricultural Industry. J. Crop Improv..

[26] Benzon H.R.L., Lee S.C. (2016). Potential of Wood Vinegar in Enhancing Fruit Yield and Antioxidant Capacity in Tomato. Korean J. Plant Resour..

[27] Dominguez F., Cejudo F.J. (2021). Chlorophyll Dismantling in Leaf Senescence. J. Exp. Bot..

[28] Tan S., Sha Y., Sun L., Li Z. (2023). Abiotic Stress-Induced Leaf Senescence: Regulatory Mechanisms and Application. Int. J. Mol. Sci..

[29] Ofoe R., Gunupuru L.R., Abbey L. (2022). Metabolites, Elemental Profile and Chemical Activities of *Pinus Strobus* High Temperature-Derived Pyroligneous Acid. Chem. Biol. Technol. Agric..

[30] Meyer S., Cerovic Z., Goulas Y., Montpied P., Demontes-Mainard S., Bidel L., Moya I., Dreyer E. (2006). Relationships between Optically Assessed Polyphenols and Chlorophyll Contents, and Leaf Mass per Area Ratio in Woody Plants: A Signature of the Carbon–Nitrogen Balance within Leaves?. Plant Cell Environ..

[31] Liu X., Li J., Cui X., Ji D., Xu Y., Chen T., Tian S. (2020). Exogenous Bamboo Pyroligneous Acid Improves Antioxidant Capacity and Primes Defense Responses of Harvested Apple Fruit. LWT.

[32] Rivera F.R., Valida A.D., Alcedo A.L. (2015). Effects of Wood Vinegar on Tomato Fruit Quality and Shelf Life at Ambient and Low Temperatures. Acta Hortic..

[33] Kaur G., Asthir B. (2015). Proline: A Key Player in Plant Abiotic Stress Tolerance. Biol. plant..

[34] Lada R.R., MacDonald M.T. (2015). Understanding the Physiology of Postharvest Needle Abscission in Balsam Fir. Front. Plant Sci..

[35] Cândido N.R., Pasa V.M.D., de Oliveira Vilela A., Campos Â D., de Fátima Â., Modolo L.V. (2023). Understanding the Multifunctionality of Pyroligneous Acid from Waste Biomass and the Potential Applications in Agriculture. Sci. Total Environ..

[36] Yang J.-F., Yang C.-H., Liang M.-T., Gao Z.-J., Wu Y.-W., Chuang L.-Y. (2016). Chemical Composition, Antioxidant, and Antibacterial Activity of Wood Vinegar from Litchi Chinensis. Molecules.

[37] Fuhrer J. (1985). Ethylene Production and Premature Senescence of Needles from Fir Trees (*Abies alba*). Eur. J. For. Pathol..

[38] Yamanaka K. (1984). Leaf Abscission and Ethylene Production in Chamaecyparis Obtusa S. et Z. J. Jpn. For. Soc..

[39] Sedigheh H.G., Mortazavian M., Norouzian D., Atyabi M., Akbarzadeh A., Hasanpoor K., Ghorbani M. (2011). Oxidative Stress and Leaf Senescence. BMC Res. Notes.

[40] Zhang Y., Wang A., Li J., Wu J. (2024). Water Content Estimation of Conifer Needles Using Leaf-Level Hyperspectral Data. Front. Plant Sci..

[41] Lichtenthaler H.K. (1987). Chlorophylls and Carotenoids: Pigments of Photosynthetic Membranes. Methods in Enzymology.

[42] Ainsworth E.A., Gillespie K.M. (2007). Estimation of Total Phenolic Content and Other Oxidative Substrates in Plant Tissues Using Folin-Ciocalteu Reagent. Nat. Protoc..

[43] Chang C.C., Yang M.H., Wen H.M., Chern J.C. (2002). Estimation of Total Flavanoid Content in Propolis by Two Complimentary Colorimetric Methods. J. Food Drug Anal..

[44] Patterson B.D., MacRae E.A., Ferguson I.B. (1984). Estimation of Hydrogen Peroxide in Plant Extracts Using Titanium(IV). Anal. Biochem..

